# Association of Serum 25-Hydroxyvitamin D Concentrations With All-Cause and Cause-Specific Mortality Among Adult Patients With Existing Cardiovascular Disease

**DOI:** 10.3389/fnut.2021.740855

**Published:** 2021-09-23

**Authors:** Lei Dai, Man Liu, Liangkai Chen

**Affiliations:** ^1^Division of Cardiology, Department of Internal Medicine, Tongji Hospital, Tongji Medical College, Huazhong University of Science and Technology, Wuhan, China; ^2^Department of Urology, Tongji Hospital, Tongji Medical College, Huazhong University of Science and Technology, Wuhan, China; ^3^Hubei Key Laboratory of Food Nutrition and Safety, Department of Nutrition and Food Hygiene, School of Public Health, Tongji Medical College, Huazhong University of Science and Technology, Wuhan, China; ^4^Ministry of Education Key Laboratory of Environment and Health, School of Public Health, Tongji Medical College, Huazhong University of Science and Technology, Wuhan, China

**Keywords:** vitamin D, cardiovascular disease, mortality, cohort study, UK Biobank

## Abstract

**Background:** Vitamin D insufficiency and deficiency are common in patients with cardiovascular disease (CVD). We aimed to prospectively examine the associations of serum 25-hydroxyvitamin D [25(OH)D] concentrations with all-cause and cause-specific mortality among adult patients with existing CVD.

**Methods:** We included 37,079 patients with CVD from the UK Biobank study, a prospective cohort of half a million participants aged 40–69 years. We defined patients with CVD as those who suffered coronary heart disease, atrial fibrillation, heart failure, or stroke. The associations of serum 25(OH)D concentration with all-cause and cause-specific mortality were examined by using multivariable Cox regression models and competing risk analyses.

**Results:** Among 37,079 patients with CVD at baseline, 57.5% were subjected to vitamin D deficiency (i.e., 25[OH]D <50 nmol/L). During a median follow-up of 11.7 years, 6,319 total deaths occurred, including 2,161 deaths from CVD, 2,230 deaths from cancer, 623 deaths from respiratory disease, and 1,305 other-cause deaths. We observed non-linear inverse associations for all-cause, cancer, respiratory disease, and other-cause mortality (*P*-non-linearity <0.01) and approximately linear inverse associations for CVD mortality (*P*-non-linearity = 0.074). Among CVD patients with vitamin D deficiency, per 10 nmol/L increment in serum 25(OH)D concentrations was associated with an 12% reduced risk for all-cause mortality and 9% reduced risk for CVD mortality.

**Conclusion:** Among patients with existing CVD, increasing levels in serum 25(OH)D were independently associated with a decreased risk of all-cause and cause-specific mortality. These findings suggest that elevated serum 25(OH)D concentration benefits CVD patients with vitamin D deficiency.

## Introduction

Cardiovascular disease (CVD) remains the leading cause of mortality and poses significant health burdens globally (1). The past few decades have witnessed an escalating research interest in the potential role of vitamin D in CVD prevention (2, 3). Multiple cardiovascular protective mechanisms of vitamin D have been proposed, including suppression of the renin-angiotensin-aldosterone system, increased insulin sensitization, anti-inflammatory actions, inhibition of foam cell formation, and parathyroid hormone (PTH) synthesis (2, 3).

Observational studies have linked low 25-hydroxyvitamin D (25[OH]D) serum levels with increased risk of subsequent CVD (3–8). Despite the accumulating mechanism- and population-based evidence of vitamin D on preventing CVD, recent randomized controlled trials (RCTs) failed to establish the cardiovascular benefits of vitamin D supplementation in the general population or those without a previous history of CVD (9–11). The null findings may be explained by the short-term duration of follow-up, lower event rate than expected, and optimal baseline 25(OH)D levels of population. Although results from these trials are not encouraging, vitamin D status remains an important worldwide public-health concern (12). Vitamin D insufficiency and deficiency are common in patients with CVD (13, 14). Existing evidence from RCTs indicates that vitamin D supplementation exerts beneficial effects on coronary artery disease (CAD) (15), left ventricular structure and function in patients with chronic heart failure (CHF) (16), and inflammatory milieu in patients with CHF (17). However, it does not reduce mortality in patients with advanced heart failure (HF) (18) and does not improve functional capacity or quality of life in older HF patients with vitamin D insufficiency (19).

Evidence from observational studies is limited and inconsistent in this regard (20–27). Prior cohort studies among patients with CVD (20), HF (21–23), suspected coronary artery disease (24), or suspected stable angina pectoris (25) found that plasma 25(OH)D concentrations are inversely associated with all-cause and cardiovascular mortality. However, two cohort studies did not find a significant association between vitamin D levels and all-cause mortality or secondary cardiovascular event incidence (26, 27). Meanwhile, previous studies are subjected to several limitations, including a relatively small sample size, ignoring dietary factors, physical activity, season of vitamin D assessment, and CVD duration and other confounding factors, which might limit the interpretation of the results. Improved understanding of the association between vitamin D status and mortality may shed new light on the potential of vitamin supplementation to provide clinical benefits for patients with CVD in further RCTs. To fill the knowledge gap, we aimed to prospectively examine the associations of serum 25(OH)D concentrations with all-cause and cause-specific mortality among adult patients with existing CVD in a large cohort.

## Methods

### Study Population

The UK Biobank is a prospective cohort of half a million participants, aged 40–69 years, recruited between 2006 and 2010 throughout the UK (28). These participants completed extensive touch screen questionnaires, physical and functional measures, and collection of biological samples. Follow-up was conducted through linkages to routinely available national datasets. Ethical approval was obtained by the National Health Service National Research Ethics Service (11/NW/0382) and renewed by the North West–Haydock Research Ethics Committee (16/NW/0274). All participants provided informed written consent. The background information about UK Biobank and details is available on the website http://www.ukbiobank.ac.uk.

Definitions of baseline CVD were constructed using the 9th revision of the International Statistical Classification of Diseases (ICD-9), ICD-10, and Office of Population, Censuses, and Surveys-4 codes as well as self-reported data fields with choice-, disease- or procedure-specific codes. We defined patients with CVD as those who suffered coronary heart disease (CHD), atrial fibrillation (AF), HF, or stroke. Detailed definition is described in Supplementary Table 1. In the present study, we excluded participants without CVD at baseline (*n* = 461,625), leaving 40,849 participants with pre-existing CVD. Participants with missing data on serum 25(OH)D (*n* = 3,760) and pregnancy (*n* = 10) were further excluded from analyses. Finally, 37,079 patients with CVD were included (Supplementary Figure 1).

### Assessment of Vitamin D Status

Vitamin D status was classified according to the serum levels of 25(OH)D. The Endocrine Society Clinical Practice Guidelines define vitamin D severe deficiency as serum 25(OH)D levels <25.0 nmol/L, moderate deficiency as 25.0–49.9 nmol/L, insufficient as 50.0–74.9 nmol/L, and sufficient as ≥75.0 nmol/L (29). In the UK biobank, analysis of serum 25(OH)D concentrations utilized immunoassay analyzers (DiaSorin Liaison XL Analyzer, made in Diasorin S.p.A) by a direct competitive chemiluminescent immunoassay method, with an analytical range of 10–375 nmol/L. A rigorous protocol was adopted to verify the assay and analyzer performance by the following parameters: precision, accuracy (or recovery) and bias, linearity, and reportable range, including the limit of quantification, carryover, and multi-instrument comparison. Additional details of UK Biobank Biomarker Project have been described in the UK Biobank Showcase (http://biobank.ndph.ox.ac.uk/showcase/showcase/docs/serum_biochemistry.pdf).

### Assessment of Outcomes

In the UK Biobank study, mortality data of each participant were obtained by linkages to National Health Service (NHS) datasets, including the NHS Digital (for England and Wales) and the NHS Central Register (for Scotland). The date of death and the causes of death were provided and coded using the ICD-10 system. In addition to the all-cause mortality, primary cause of death was extracted from the UK Biobank Cause of Death Registry, including cardiovascular mortality (ICD-10: I00-I99), cancer mortality (ICD-10: C00-C97), respiratory disease mortality (ICD-10: J00-J99), and other-cause mortality (mortality excluding cardiovascular, cancer, and respiratory disease).

### Covariates

Information on age, sex, ethnicity, education, household income, and lifestyle behaviors was acquired using touch screen questionnaires. Participants were labeled as never, former, and current smokers according to the summarized smoking status (Field ID: 20116). Daily average alcohol consumption was described depending on the drinking frequency and the number of drink-equivalents/day. Physical activity was classified in accordance with 2018 Physical Activity Guidelines for Americans as inactive (those with no documented leisure time physical activity), insufficient (<150 min/week of moderate activity and <75 min/week of vigorous activity), and active (≥150 min/week of moderate activity and/or ≥75 min/week of vigorous activity) (30). We constructed a healthy diet score with reference to the dietary priorities for cardiometabolic health recommended by American Heart Association (31). Definitions of each component of a healthy diet score are shown in Supplementary Table 2. The scale of the healthy diet score ranged from 0 to 10, and a higher score equates to a much healthier dietary pattern. Adherence to a healthy diet was defined as participants who had at least 5 scores of healthy diet components. Socioeconomic status was indicated by Townsend deprivation index scores, and higher Townsend scores equate to higher levels of socioeconomic deprivation (32). Details regarding health conditions and drug use were ascertained by touch screen questionnaires, face-to-face interviews, and linkage to electronic health records. A trained nurse measured the height, weight, and blood pressure during the initial assessment. Serum was collected through venipuncture, and biomarkers including glycated hemoglobinA1c (HbA1c), lipids, and C-reactive protein (CRP) were measured. The estimated glomerular filtration rate (creatinine-cystatin C equation, eGFRcr-cys) was calculated from serum creatinine and cystatin C (33). Further details of these measurements are available on the UK Biobank website (http://www.ukbiobank.ac.uk).

### Statistical Analysis

Multivariable Cox regression models to the estimate hazard ratios (HRs) and 95% confidence intervals (CIs) were used for the associations of the serum 25(OH)D concentration with all-cause mortality. Competing risk analyses were conducted using the cause-specific hazard function model to estimate hazards for CVD, cancer, respiratory disease, and other-cause mortality (34, 35). The time to events was calculated from the date of the blood sample collection to the death or the censoring date (31, December, 2020), whichever came first. Participants with severe deficiency of vitamin D (serum 25[OH]D <25.0 nmol/L) were selected as the reference group. Models were successively adjusted for age (continuous), sex (male, female), ethnicity (White, mixed, Asian, Black, Chinese, others), education (college or university, vocational qualification, upper secondary, lower secondary, others), Townsend deprivation index (in quintiles), household income (<18,000; 18,000–30,999; 31,000–51,999; 52,000–100,000; >1,00,000 £), smoking status (never smoker, former smoker, current smoker), alcohol consumption (0, 0.1–4.9, 5.0–14.9, 15.0–19.9, 20.0–29.9, ≥30.0 g/day), physical activity (inactive, insufficient, active), healthy diet score (in quintiles), BMI (<18.5, 18.5–22.9, 23.0–24.9, 25.0–29.9, 30.0–34.9, ≥35.0 kg/m^2^), eGFR_cr−cys_ (<30.0, 30.0–60.0, 60.0–90.0, ≥90.0 mL min^−1^ per 1.73 m^2^), CRP (in quintiles), anti-hypertensive medication use, cholesterol lowering medication use, diabetes medication use (none, only oral medication, only insulin, or insulin and oral medication), history of cancer, diabetes, hypertension, and CVD duration (<1.0, 1.0–4.9, 5.0–9.9, ≥10.0 years). Missing values of covariates were treated as dummy variables. The dose-response curves presenting the hazard of serum 25(OH)D were fitted by using the restricted cubic spline model with four knots (rms, hmisc, lattice, and survival packages in R software).

Several sensitivity analyses were performed to test the robustness of our results. We performed subgroup analyses across age, sex, BMI, smoking status, physical activity, dietary supplement use, antihypertensive treatment, cholesterol lowering medication, and CVD duration. The joint test was used to obtain a *P*-value for interaction for examining the statistical significance of the difference between subgroups. Considering the influence of seasonal fluctuations and time spent outdoors on circulating 25(OH)D concentrations, we further adjusted for the month of blood collection (January through December, categorical), time spent outdoors in summer (continuous), and time spent outdoors in winter (continuous) in Cox regression models. We further adjusted for blood pressure, HbA1c, lipids, and dietary supplement use, including vitamin D supplements, multivitamin supplements, mineral supplements, fish oil, and glucosamine. We also excluded patients with diagnosed thyroid and parathyroid diseases. In view of the unavailable data on blood parathyroid hormone levels in UK Biobank, we adjusted for the serum levels of calcium and phosphate (36). We also considered energy intake and dietary vitamin D intake of 12,505 patients who completed 24h dietary recalls between April 2009 and June 2012. To minimize the potential reverse causation bias, we excluded patients who died within 4 years and re-examined the association between serum 25(OH)D and mortality.

Statistical analyses were performed between 1 November, 2020 and 13 July, 2021. SAS version 9.4 (SAS Institute, USA) and R software (The R Foundation, http://www.r-project.org, version 4.0.2) were utilized for analyses and plotting with a two-sided significance threshold of *P* < 0.05.

## Results

### Baseline Characteristics

A total of 37,079 patients with CVD (mean age, 61.4 years [SD, 6.4 years]; 12,668 [34.2%] females) were included in the present analysis. Table 1 shows the baseline characteristics according to serum 25(OH)D levels. Among these patients, 5,773 (15.6%); 15,557 (42.0%); 11,451 (30.9%); and 4,298 (11.6%) were in the vitamin D status of severe deficiency, moderate deficiency, insufficient, and sufficient, respectively. Compared with patients with vitamin D deficiency, those with higher serum 25(OH)D concentrations were more likely to be older, male, non-smoker, and to have lower levels of socioeconomic deprivation, drink more alcohol, adhere to a healthy diet pattern, exercise more, use more dietary supplements, have a lower BMI, and have lower prevalence of hypertension and diabetes but a higher prevalence of cancer.

**Table 1:** Baseline characteristics of 37,079 patients with cardiovascular disease in UK Biobank.

**Characteristics**	**Serum 25(OH)D concentrations, nmol/L**			
	** <25 nmol/L** **(*n* = 5,773)**	**25–49.9 nmol/L** **(*n* = 15,557)**	**50–74.9 nmol/L** **(*n* = 11,451)**	**≥75 nmol/L** **(*n* = 4,298)**
Age, mean (SD), years	59.8 (7.0)	61.2 (6.4)	62.1 (6.0)	62.2 (6.0)
Male, *n* (%)	3,604 (62.4%)	10,101 (64.9%)	7,754 (67.7%)	2,952 (68.7%)
Socioeconomic status, median (IQR)	0.2 (−2.6 to 3.3)	−1.4 (−3.3 to 1.8)	−2.2 (−3.7 to 0.4)	−2.4 (−3.7 to −0.1)
**Education**, ***n*** **(%)**				
College or university	1,244 (21.5%)	3,569 (22.9%)	2,513 (21.9%)	934 (21.7%)
Vocational qualification	750 (13.0%)	2,233 (14.4%)	1,717 (15.0%)	628 (14.6%)
Upper secondary	527 (9.1%)	1,316 (8.5%)	982 (8.6%)	350 (8.1%)
Lower secondary	1,239 (21.5%)	3,444 (22.1%)	2,585 (22.6%)	1,039 (24.2%)
Others	1,880 (32.6%)	4,717 (30.3%)	3,463 (30.2%)	1,272 (29.6%)
Unknown	133 (2.3%)	278 (1.8%)	191 (1.7%)	75 (1.7%)
**Ethnicity**, ***n*** **(%)**				
White	4,946 (85.7%)	14,675 (94.3%)	11,179 (97.6%)	4,247 (98.8%)
Mixed	47 (0.8%)	78 (0.5%)	24 (0.2%)	7 (0.2%)
Asian	464 (8.0%)	323 (2.1%)	79 (0.7%)	16 (0.4%)
Black	154 (2.7%)	240 (1.5%)	66 (0.6%)	12 (0.3%)
Chinese	19 (0.3%)	26 (0.2%)	10 (0.1%)	0 (0.0%)
Others	97 (1.7%)	131 (0.8%)	45 (0.4%)	5 (0.1%)
Unknown	46 (0.8%)	84 (0.5%)	48 (0.4%)	11 (0.3%)
**Household income, £**				
<18,000	2,295 (39.8%)	5,051 (32.5%)	3,446 (30.1%)	1,232 (28.7%)
18,000–30,999	1,090 (18.9%)	3,533 (22.7%)	2,767 (24.2%)	1,061 (24.7%)
31,000–51,999	725 (12.6%)	2,366 (15.2%)	1,960 (17.1%)	744 (17.3%)
52,000–100,000	434 (7.5%)	1,417 (9.1%)	1,054 (9.2%)	419 (9.7%)
>100,000	89 (1.5%)	357 (2.3%)	273 (2.4%)	131 (3.0%)
Unknown	1,140 (19.7%)	2,833 (18.2%)	1,951 (17.0%)	711 (16.5%)
**Smoking status**, ***n*** **(%)**				
Never smoker	2,119 (36.7%)	6,342 (40.8%)	4,843 (42.3%)	1,796 (41.8%)
Former smoker	2,356 (40.8%)	7,168 (46.1%)	5,564 (48.6%)	2,154 (50.1%)
Current smoker	1,238 (21.4%)	1,947 (12.5%)	935 (8.2%)	312 (7.3%)
Unknown	60 (1.0%)	100 (0.6%)	109 (1.0%)	36 (0.8%)
Alcohol consumption, median (IQR), g/day	8.0 (0–23.1)	10.2 (2.2–22.4)	11.9 (3.6–23.0)	13.0 (5.1–25.5)
Adherence to a healthy diet^*^, *n* (%)	1,130 (19.6%)	3,118 (20.0%)	2,501 (21.8%)	981 (22.8%)
**Physical activity**, ***n*** **(%)**				
Inactive group	1,619 (28.0%)	3,360 (21.6%)	1,850 (16.2%)	640 (14.9%)
Insufficient group	1,617 (28.0%)	4,136 (26.6%)	2,876 (25.1%)	960 (22.3%)
Active group	1,903 (33.0%)	6,738 (43.3%)	5,935 (51.8%)	2,457 (57.2%)
Unknown	634 (11.0%)	1,323 (8.5%)	790 (6.9%)	241 (5.6%)
**Dietary supplement use**, ***n*** **(%)**				
Vitamin D supplements	104 (1.8%)	434 (2.8%)	517 (4.5%)	261 (6.1%)
Multivitamin supplements	489 (8.5%)	2,262 (14.5%)	2,508 (21.9%)	1,049 (24.4%)
Mineral supplements	472 (8.2%)	1,406 (9.0%)	1,477 (12.9%)	711 (16.5%)
Fish oil	926 (16.0%)	4,302 (27.7%)	4,701 (41.1%)	1,935 (45.0%)
Glucosamine	470 (8.1%)	2,065 (13.3%)	2,250 (19.6%)	889 (20.7%)
BMI, mean (SD), kg/m^2^	30.4 (6.2)	29.6 (5.2)	28.5 (4.5)	27.4 (4.0)
Antihypertensive medication, *n* (%)	3,638 (63.0%)	9,522 (61.2%)	6,767 (59.1%)	2,517 (58.6%)
Cholesterol lowering medication, *n* (%)	4,173 (72.3%)	11,084 (71.2%)	8,162 (71.3%)	3,217 (74.8%)
**Diabetes medication use**, ***n*** **(%)**				
None	4,747 (82.2%)	13,590 (87.4%)	10,480 (91.5%)	4,001 (93.1%)
Only oral medication	668 (11.6%)	1,298 (8.3%)	687 (6.0%)	191 (4.4%)
Only insulin	154 (2.7%)	285 (1.8%)	124 (1.1%)	59 (1.4%)
Insulin and oral medication	204 (3.5%)	384 (2.5%)	160 (1.4%)	47 (1.1%)
Hypertension, *n* (%)	4,823 (83.5%)	12,736 (81.9%)	9,093 (79.4%)	3,397 (79.0%)
Diabetes, *n* (%)	1,507 (26.1%)	2,981 (19.2%)	1,561 (13.6%)	471 (11.0%)
Cancer, *n* (%)	733 (12.7%)	2,024 (13.0%)	1,579 (13.8%)	633 (14.7%)
**Duration of CVD, years**, ***n*** **(%)**				
<1	613 (10.6%)	1,879 (12.1%)	1,459 (12.7%)	528 (12.3%)
1–4.9	1,699 (29.4%)	4,735 (30.4%)	3,593 (31.4%)	1,377 (32.0%)
5–9.9	1,583 (27.4%)	4,245 (27.3%)	3,075 (26.9%)	1,156 (26.9%)
≥10	1,878 (32.5%)	4,698 (30.2%)	3,324 (29.0%)	1,237 (28.8%)
eGFRcr-cys, mean (SD), ml min^−1^ per 1.73 m^2^	81.5 (17.9)	81.8 (16.4)	82.1 (15.1)	82.2 (15.2)
C-reactive protein, median (IQR), mg/L	2.1 (1.0–4.5)	1.7 (0.8–3.5)	1.5 (0.7–3.0)	1.3 (0.6–2.6)

*Data were expressed as the mean (SD), median (IQR), or n (%)*.

* *Adherence to a healthy diet was defined as participants who had at least 5 score of healthy diet components*.

*BMI, body mass index; eGFRcr-cys, estimated glomerular filtration rate (creatinine–cystatin C equation); CVD, cardiovascular disease; IQR, interquartile range; SD, standard deviation*.

The adjusted means (95% CIs) of cardiometabolic markers were presented according to serum 25(OH)D concentrations (Table 2). Higher levels of serum 25(OH)D were significantly associated with lower levels of triglycerides, total cholesterol, low-density lipoprotein direct, lipoprotein(a), apolipoprotein-A, apolipoprotein-B, systolic blood pressure, diastolic blood pressure, HbA1c, CRP, and eGFRcr-cys (all *P*_trend_ < 0.05). However, levels of high-density lipoprotein cholesterol did not change significantly across serum 25(OH)D categories (*P*_trend_ = 0.721).

**Table 2:** Adjusted means of cardiometabolic markers according to serum 25(OH)D concentrations among 37,079 CVD patients.

**Characteristics**	**Serum 25(OH)D concentrations, nmol/L**				***P*_**trend**_**
	** <25.0 nmol/L** **(*n* = 5,773)**	**25.0–49.9 nmol/L** **(*n* = 15,557)**	**50.0–74.9 nmol/L** **(*n* = 11,451)**	**≥75.0 nmol/L** **(*n* = 4,298)**	
Triglycerides, mg/dL	185.6 (183.2, 187.9)	171.4 (170.0, 172.8)	159.2 (157.6, 160.9)	138.7 (136.0, 141.4)	<0.001
Total cholesterol, mg/dL	181.0 (180.2, 181.9)	176.4 (175.9, 176.9)	173.5 (172.9, 174.1)	166.5 (165.5, 167.5)	<0.001
High-density lipoprotein cholesterol, mg/dL	47.2 (46.9, 47.4)	46.5 (46.4, 46.7)	46.8 (46.6, 47.0)	46.9 (46.5, 47.2)	0.721
Low-density lipoprotein cholesterol, mg/dL	109.9 (109.2, 110.5)	107.3 (106.9, 107.7)	105.3 (104.9, 105.8)	100.2 (99.5, 101.0)	<0.001
Lipoprotein(a), nmol/L	47.9 (46.3, 49.5)	48.9 (47.9, 49.8)	47.3 (46.2, 48.3)	46.4 (44.6, 48.2)	0.048
Apolipoprotein-A, g/L	1.45 (1.44, 1.46)	1.44 (1.43, 1.44)	1.44 (1.44, 1.44)	1.43 (1.42, 1.43)	<0.001
Apolipoprotein-B, g/L	0.92 (0.91, 0.92)	0.90 (0.89, 0.90)	0.88 (0.88, 0.89)	0.84 (0.84, 0.85)	<0.001
Systolic blood pressure, mmHg	139.6 (139.1, 140.0)	138.9 (138.7, 139.2)	138.6 (138.3, 138.9)	137.7 (137.2, 138.2)	<0.001
Diastolic blood pressure, mmHg	80.9 (80.7, 81.2)	80.3 (80.1, 80.5)	80.3 (80.1, 80.4)	79.7 (79.4, 80.0)	<0.001
HbA1c, mmol/mol	40.0 (39.8, 40.2)	39.5 (39.4, 39.6)	39.2 (39.1, 39.4)	39.0 (38.8, 39.2)	<0.001
C-reactive protein, mg/L	3.5 (3.4, 3.7)	3.2 (3.1, 3.2)	3.1 (3.0, 3.2)	3.2 (3.0, 3.3)	<0.001
eGFRcr-cys, ml min^−1^ per 1.73 m^2^	81.9 (81.6, 82.3)	82.0 (81.8, 82.2)	82.0 (81.7, 82.2)	81.2 (80.8, 81.6)	0.03

*Data were expressed as the adjusted mean (95% CI) and adjusted for age (continuous), sex (male, female), ethnicity (White, mixed, Asian, Black, Chinese, others, or unknown), education (college or university, vocational qualification, upper secondary, lower secondary, others, or unknown), Townsend deprivation index (in quintiles), household income (<18,000, 18,000–30,999, 31,000–51,999, 52,000–1,00,000, >1,00,000 £, or unknown), smoking status (never smoker, former smoker, current smoker, or unknown), alcohol consumption (0, 0.1–4.9, 5.0–14.9, 15.0–19.9, 20.0–29.9, ≥30.0 g/day, or unknown), physical activity (inactive group, insufficient group, active group, or unknown), healthy diet score (in quintiles), BMI (<18.5, 18.5–22.9, 23.0–24.9, 25.0–29.9, 30.0–34.9, or ≥35.0 kg/m^2^), antihypertensive medication use (yes, no), cholesterol lowering medication use (yes, no), diabetes medication use (none, only oral medication, only insulin, or insulin and oral medication), history of cancer, diabetes, hypertension, and duration of CVD (<1.0, 1.0–4.9, 5.0–9.9, or ≥10.0 years). CVD, cardiovascular disease; eGFRcr-cys, estimated glomerular filtration rate (creatinine–cystatin C equation); HbA1C, glycated hemoglobinA1c*.

### Associations of Serum 25(OH)D Concentrations With All-Cause and Cause-Specific Mortality

During 412,046 person-years of follow-up (median 11.7 years), 6,319 total deaths occurred, including 2,161 deaths from CVD, 2,230 deaths from cancer, 623 deaths from respiratory disease, and 1,305 other-cause deaths. In Cox regression analyses, compared with patients in severe deficiency of vitamin D (serum 25[OH]D <25 nmol/L), those with moderate deficiency, insufficient, or sufficient vitamin D status showed a decreased hazard for all-cause and cause-specific mortality (Table 3). These associations remained robust after stepwise adjustment for confounders. In the fully adjusted model, the HRs and 95% CIs from the lowest to the highest serum 25(OH)D categories (<25.0, 25.0–49.9, 50.0–74.9, and ≥75.0 nmol/L) were 1.00 (reference), 0.78 (0.73, 0.84), 0.70 (0.65, 0.76), and 0.66 (0.59, 0.73), respectively, for all-cause mortality; 1.00 (reference), 0.79 (0.71, 0.89), 0.71 (0.63, 0.81), and 0.59 (0.49, 0.70), respectively, for CVD mortality; 1.00 (reference), 0.88 (0.79, 0.995), 0.78 (0.69, 0.89), and 0.77 (0.65, 0.91), respectively, for cancer mortality; 1.00 (reference), 0.72 (0.59, 0.89), 0.58 (0.46, 0.74), and 0.64 (0.47, 0.87), respectively, for respiratory disease mortality; and 1.00 (reference), 0.67 (0.58, 0.77), 0.64 (0.54, 0.75), and 0.62 (0.50, 0.77), respectively, for other-cause mortality (Model 3 in Table 3).

**Table 3:** Associations of serum 25(OH)D concentrations with all-cause and cause-specific mortality among 37,079 CVD patients.

	**Serum 25(OH)D concentrations, nmol/L**				**Per 10 nmol/L increment in 25(OH)D**	
	** <25.0 nmol/L** **(*n* = 5,773)**	**25.0–49.9 nmol/L** **(*n* = 15,557)**	**50.0–74.9 nmol/L** **(*n* = 11,451)**	**≥75.0 nmol/L** **(*n* = 4,298)**	**Patients with** ** 25(OH)D <50.0 nmol/L** **(*n* = 21,331)**	**Patients with** ** 25(OH)D ≥50.0 nmol/L** **(*n* = 15,749)**
**All-cause mortality**						
Incident rate per 1,000 person-year (cases)	21.99 (1,368)	15.55 (2,687)	13.04 (1,679)	12.11 (585)	17.26 (4,055)	12.79 (2,264)
Model 1	1 (ref.)	0.61 (0.58, 0.66)	0.47 (0.44, 0.51)	0.43 (0.39, 0.48)	0.78 (0.76, 0.80)	0.97 (0.94, 0.996)
Model 2	1 (ref.)	0.75 (0.70, 0.80)	0.65 (0.60, 0.70)	0.61 (0.56, 0.68)	0.86 (0.83, 0.89)	0.98 (0.95, 1.01)
Model 3	1 (ref.)	0.78 (0.73, 0.84)	0.70 (0.65, 0.76)	0.66 (0.59, 0.73)	0.88 (0.85, 0.90)	0.98 (0.95, 1.002)
**Cardiovascular disease mortality**						
Incident rate per 1,000 person-year (cases)	7.76 (483)	5.41 (935)	4.44 (571)	3.56 (172)	6.03 (1,418)	4.20 (743)
Model 1	1 (ref.)	0.61 (0.55, 0.68)	0.46 (0.41, 0.52)	0.37 (0.31, 0.44)	0.80 (0.76, 0.84)	0.92 (0.88, 0.97)
Model 2	1 (ref.)	0.75 (0.67, 0.84)	0.64 (0.56, 0.72)	0.53 (0.44, 0.64)	0.88 (0.84, 0.93)	0.94 (0.89, 0.99)
Model 3	1 (ref.)	0.79 (0.71, 0.89)	0.71 (0.63, 0.81)	0.59 (0.49, 0.70)	0.91 (0.86, 0.96)	0.93 (0.89, 0.98)
**Cancer mortality**						
Incident rate per 1,000 person-year (cases)	6.65 (414)	5.57 (962)	4.84 (623)	4.78 (231)	5.86 (1,376)	4.82 (854)
Model 1	1 (ref.)	0.71 (0.64, 0.80)	0.57 (0.50, 0.65)	0.55 (0.47, 0.65)	0.83 (0.78, 0.87)	0.99 (0.95, 1.04)
Model 2	1 (ref.)	0.86 (0.77, 0.97)	0.75 (0.66, 0.86)	0.74 (0.63, 0.88)	0.90 (0.85, 0.95)	1.00 (0.96, 1.04)
Model 3	1 (ref.)	0.88 (0.79, 0.995)	0.78 (0.69, 0.89)	0.77 (0.65, 0.91)	0.90 (0.86, 0.95)	1.00 (0.95, 1.04)
**Respiratory disease mortality**						
Incident rate per 1,000 person-year (cases)	2.60 (162)	1.51 (261)	1.10 (141)	1.22 (59)	1.80 (423)	1.13 (200)
Model 1	1 (ref.)	0.50 (0.41, 0.61)	0.33 (0.27, 0.42)	0.37 (0.27, 0.50)	0.68 (0.62, 0.75)	1.06 (0.98, 1.15)
Model 2	1 (ref.)	0.69 (0.57, 0.85)	0.53 (0.42, 0.67)	0.59 (0.43, 0.81)	0.80 (0.72, 0.88)	1.07 (0.98, 1.16)
Model 3	1 (ref.)	0.72 (0.59, 0.89)	0.58 (0.46, 0.74)	0.64 (0.47, 0.87)	0.81 (0.74, 0.90)	1.06 (0.97, 1.15)
**Other mortality**						
Incident rate per 1,000 person-year (cases)	4.97 (309)	3.06 (529)	2.67 (344)	2.55 (123)	3.57 (838)	2.64 (467)
Model 1	1 (ref.)	0.54 (0.47, 0.63)	0.44 (0.38, 0.51)	0.42 (0.34, 0.51)	0.73 (0.68, 0.78)	0.96 (0.90, 1.02)
Model 2	1 (ref.)	0.65 (0.56, 0.75)	0.58 (0.50, 0.69)	0.57 (0.46, 0.71)	0.80 (0.74, 0.85)	0.96 (0.90, 1.02)
Model 3	1 (ref.)	0.67 (0.58, 0.77)	0.64 (0.54, 0.75)	0.62 (0.50, 0.77)	0.81 (0.76, 0.87)	0.95 (0.90, 1.02)

*Model 1: adjusted for age (continuous), sex (male, female), and ethnicity (White, mixed, Asian, Black, Chinese, others, or unknown)*.

*Model 2: adjusted for model 1 plus education (college or university, vocational qualification, upper secondary, lower secondary, others, or unknown), Townsend deprivation index (in quintiles), household income (<18,000, 18,000–30,999, 31,000–51,999, 52,000–1,00,000, >1,00,000 £, or unknown), smoking status (never smoker, former smoker, current smoker, or unknown), alcohol consumption (0, 0.1–4.9, 5.0–14.9, 15.0–19.9, 20.0–29.9, ≥30.0 g/day, or unknown), physical activity (inactive group, insufficient group, active group, or unknown), healthy diet score (in quintiles), and BMI (<18.5, 18.5–22.9, 23.0–24.9, 25.0–29.9, 30.0–34.9, or ≥35.0 kg/m^2^)*.

*Model 3: adjusted for model 2 plus eGFRcr-cys (<30.0, 30.0–60.0, 60.0–90.0, ≥90.0 ml min^−1^ per 1.73 m^2^), C-reactive protein (in quintiles), antihypertensive medication use (yes, no), cholesterol lowering medication use (yes, no), diabetes medication use (none, only oral medication, only insulin, or insulin and oral medication), history of cancer, diabetes, hypertension, and duration of CVD (<1.0, 1.0–4.9, 5.0–9.9, or ≥10.0 years). BMI, body mass index; CVD, cardiovascular disease; eGFRcr-cys, estimated glomerular filtration rate (creatinine–cystatin C equation)*.

**Figure 1:**
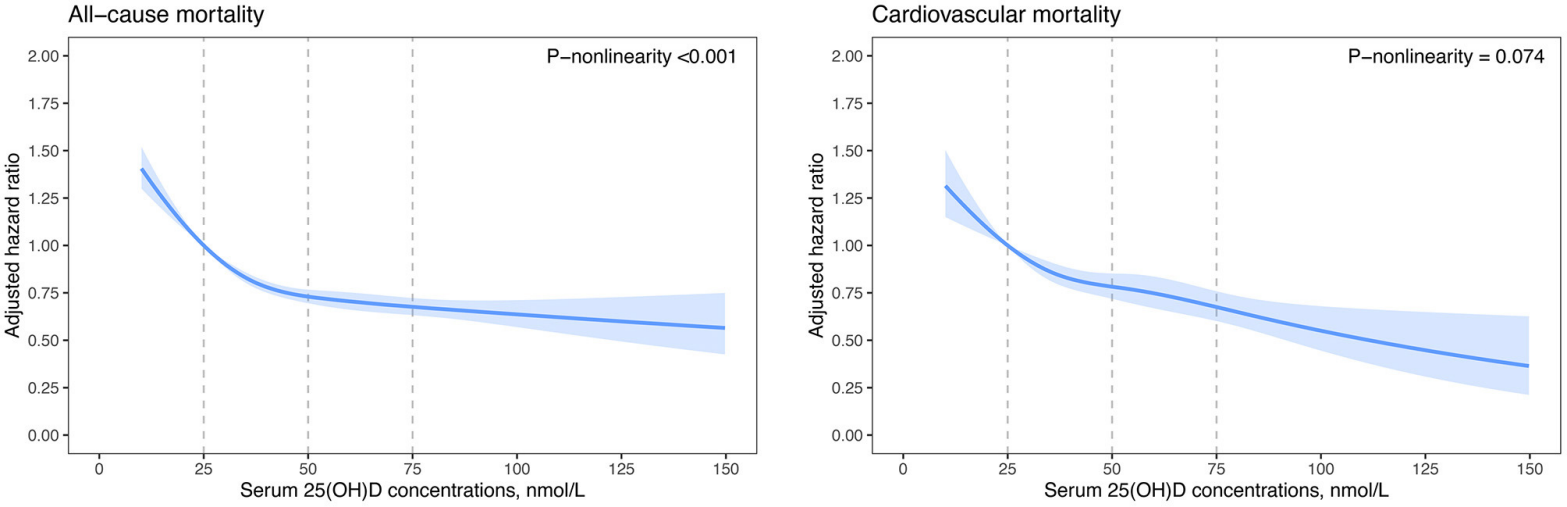
Dose-response curves for serum 25(OH)D concentrations and all-cause and cardiovascular mortality. Hazard ratios (blue lines) and 95% confidence intervals (light blue shade) were adjusted for age (continuous), sex (male, female), and ethnicity (White, mixed, Asian, Black, Chinese, others, or unknown), education (college or university, vocational qualification, upper secondary, lower secondary, others, or unknown), Townsend deprivation index (in quintiles), household income (<18,000, 18,000–30,999, 31,000–51,999, 52,000–1,00,000, >1,00,000 £, or unknown), smoking status (never smoker, former smoker, current smoker, or unknown), alcohol consumption (0, 0.1–4.9, 5.0–14.9, 15.0–19.9, 20.0–29.9, ≥30.0 g/day, or unknown), physical activity (inactive group, insufficient group, active group, or unknown), healthy diet score (in quintiles), and BMI (<18.5, 18.5–22.9, 23.0–24.9, 25.0–29.9, 30.0–34.9, or ≥35.0 kg/m^2^), eGFRcr-cys (<30.0, 30.0–60.0, 60.0–90.0, ≥90.0 ml min^−1^ per 1.73 m^2^), C-reactive protein (in quintiles), antihypertensive medication use (yes, no), cholesterol lowering medication use (yes, no), diabetes medication use (none, only oral medication, only insulin, or insulin and oral medication), history of cancer, diabetes, hypertension, and duration of CVD (<1.0, 1.0–4.9, 5.0–9.9, or ≥10.0 years). BMI, body mass index; CVD, cardiovascular disease; eGFRcr-cys, estimated glomerular filtration rate (creatinine–cystatin C equation).

We further fitted smoothing splines to present the dose-response relationship between serum 25(OH)D concentrations and risk of mortality. All-cause and CVD mortality decreased with increasing serum 25(OH)D concentrations and then reached a plateau at around 50 nmol/L 25(OH)D (Figure 1). The test of non-linearity was statistically significant (*P*-non-linearity <0.001) for all-cause mortality but not significant for CVD mortality (*P*-non-linearity = 0.074). Similar non-linear trends were observed for cancer, respiratory disease, and other-cause mortality (Supplementary Figure 2). Among patients with vitamin D deficiency (serum 25(OH)D <50.0 nmol/L), per 10 nmol/L increment in serum 25(OH)D concentrations was associated with a 12% (HR, 0.88 [95%CI: 0.85, 0.90]) reduced risk for all-cause mortality, 9% (HR, 0.91 [95%CI: 0.86, 0.95]) reduced risk for CVD mortality, 10% (HR, 0.90 [95%CI: 0.86, 0.95]) reduced risk for cancer mortality, 19% (HR, 0.81[95%CI: 0.74, 0.90]) reduced risk for respiratory disease mortality, and a 19% (HR, 0.81 [95%CI: 0.76, 0.87]) reduced risk for other-cause mortality (Table 3). However, among patients with 25(OH)D concentrations ≥50.0 nmol/L, increment in 25(OH)D concentrations was not significantly associated with a decreased risk for all-cause and cause-specific mortality, excluding CVD mortality.

We also investigated the associations of vitamin D status with all-cause, CVD, cancer, respiratory disease, and other-cause mortality of various CVD types, including CHD, AF, HF, and stroke (Table 4). Among patients with stroke, the HRs and 95% CIs from the lowest to the highest serum 25(OH)D categories were 1.00 (reference), 0.80 (0.70, 0.92), 0.69 (0.59, 0.80), and 0.59 (0.48, 0.73), respectively, for all-cause mortality and 1.00 (reference), 0.87 (0.69, 1.10), 0.73 (0.55, 0.96), and 0.45 (0.29, 0.70), respectively, for CVD mortality.

**Table 4:** Hazard ratios for all-cause and cause-specific mortality by serum 25(OH)D among patients with CVD subtypes.

		**Serum 25(OH)D concentrations, nmol/L**			
	**Incident rate per 1,000 person-year**	**<25.0 nmol/L**	**25.0–49.9 nmol/L**	**50.0–74.9 nmol/L**	**≥75.0 nmol/L**
**Coronary heart disease (*****n*** **=** **26,359)**					
All-cause mortality	15.86	1 (ref.)	0.79 (0.73, 0.85)	0.70 (0.64, 0.77)	0.67 (0.60, 0.76)
CVD mortality	5.69	1 (ref.)	0.78 (0.69, 0.89)	0.70 (0.6, 0.81)	0.59 (0.48, 0.72)
Cancer mortality	5.39	1 (ref.)	0.93 (0.81, 1.07)	0.79 (0.68, 0.93)	0.82 (0.67, 1.002)
Respiratory disease mortality	1.58	1 (ref.)	0.70 (0.56, 0.89)	0.59 (0.44, 0.77)	0.60 (0.41, 0.87)
Other-cause mortality	3.19	1 (ref.)	0.66 (0.55, 0.78)	0.65 (0.53, 0.79)	0.66 (0.51, 0.85)
**Atrial fibrillation (*****n*** **=** **6,914)**					
All-cause mortality	18.06	1 (ref.)	0.80 (0.69, 0.94)	0.76 (0.64, 0.90)	0.73 (0.58, 0.91)
CVD mortality	7.19	1 (ref.)	0.80 (0.63, 1.03)	0.78 (0.60, 1.03)	0.71 (0.50, 1.01)
Cancer mortality	5.37	1 (ref.)	0.80 (0.59, 1.08)	0.76 (0.55, 1.04)	0.75 (0.50, 1.11)
Respiratory disease mortality	1.65	1 (ref.)	0.96 (0.58, 1.60)	0.75 (0.41, 1.34)	0.99 (0.49, 2.02)
Other-cause mortality	3.84	1 (ref.)	0.76 (0.54, 1.05)	0.76 (0.53, 1.10)	0.64 (0.39, 1.04)
**Heart failure (*****n*** **=** **2,133)**					
All-cause mortality	37.05	1 (ref.)	0.78 (0.65, 0.94)	0.72 (0.58, 0.90)	0.61 (0.45, 0.82)
CVD mortality	19.12	1 (ref.)	0.78 (0.60, 1.01)	0.80 (0.59, 1.09)	0.65 (0.43, 0.996)
Cancer mortality	7.27	1 (ref.)	0.81 (0.49, 1.33)	0.57 (0.31, 1.05)	0.56 (0.27, 1.17)
Respiratory disease mortality	4.20	1 (ref.)	0.56 (0.33, 0.97)	0.61 (0.32, 1.18)	0.69 (0.30, 1.56)
Other-cause mortality	6.47	1 (ref.)	1.14 (0.72, 1.81)	0.85 (0.48, 1.50)	0.79 (0.38, 1.62)
**Stroke (*****n*** **=** **8,059)**					
All-cause mortality	17.07	1 (ref.)	0.80 (0.70, 0.92)	0.69 (0.59, 0.80)	0.59 (0.48, 0.73)
CVD mortality	5.43	1 (ref.)	0.87 (0.69, 1.10)	0.73 (0.55, 0.96)	0.45 (0.29, 0.70)
Cancer mortality	6.23	1 (ref.)	0.90 (0.72, 1.13)	0.80 (0.62, 1.03)	0.68 (0.47, 0.97)
Respiratory disease mortality	1.65	1 (ref.)	0.62 (0.40, 0.94)	0.64 (0.40, 1.04)	0.70 (0.39, 1.28)
Other-cause mortality	3.75	1 (ref.)	0.65 (0.49, 0.86)	0.52 (0.38, 0.73)	0.58 (0.38, 0.90)

*Data are presented as hazard ratio (95% confidence interval) and adjusted for age (continuous), sex (male, female), ethnicity (White, mixed, Asian, Black, Chinese, others, or unknown), education (college or university, vocational qualification, upper secondary, lower secondary, others, or unknown), Townsend deprivation index (in quintiles), household income (<18,000, 18,000–30,999, 31,000–51,999, 52,000–1,00,000, >1,00,000 £, or unknown), smoking status (never smoker, former smoker, current smoker, or unknown), alcohol consumption (0, 0.1–4.9, 5.0–14.9, 15.0–19.9, 20.0–29.9, ≥30.0 g/day, or unknown), physical activity (inactive group, insufficient group, active group, or unknown), healthy diet score (in quintiles), BMI (<18.5, 18.5–22.9, 23.0–24.9, 25.0–29.9, 30.0–34.9, or ≥35.0 kg/m^2^), eGFRcr-cys (<30.0, 30.0–60.0, 60.0–90.0, ≥90.0 ml min^−1^ per 1.73 m^2^), C-reactive protein (in quintiles), antihypertensive medication use (yes, no), cholesterol lowering medication use (yes, no), diabetes medication use (none, only oral medication, only insulin, or insulin and oral medication), history of cancer, diabetes, hypertension, and duration of CVD (<1.0, 1.0–4.9, 5.0–9.9, or ≥10.0 years). BMI, body mass index; CVD, cardiovascular disease; eGFRcr-cys, estimated glomerular filtration rate (creatinine–cystatin C equation)*.

### Sensitivity Analyses

In stratified analyses, the associations of serum 25(OH)D concentration with all-cause and CVD mortality were robust across the strata of age, sex, BMI, smoking status, physical activity, dietary supplement use, antihypertensive treatment, cholesterol lowering medication, and CVD duration (Supplementary Table 3). We further plotted smoothing splines to present the influence of season variations and outdoor exposure time on serum 25(OH)D. Serum 25(OH)D concentrations increased with prolonged time spent outdoors, and the seasonal changes in 25(OH)D concentration showed a peak during the summer months and a trough during the winter months (Supplementary Figure 3). Additional adjustment of the confounders of the month of blood collection and time spend outdoors did not change the associations between serum 25(OH)D and mortality risk (Supplementary Table 4, Model 1). Further sensitivity analyses with additional adjustment of dietary supplement use, mean arterial pressure, HbA1c, triglycerides, and high-density lipoprotein cholesterol produced similar results (Supplementary Table 4, Models 2 and 3). These associations did not change appreciably with further adjustment of serum calcium and phosphate after excluding participants with a history of thyroid or parathyroid diseases (Supplementary Table 5). In addition, we excluded participants who died within 2 years of follow-up and re-examined the associations. Results did not alter the significance of the associations between 25(OH)D concentration and all-cause and cause-specific mortality (Supplementary Table 6). Similar results were also obtained with further adjustment of energy intake and dietary vitamin D intake (Supplementary Table 7).

## Discussion

In this large prospective cohort study, near 60% of patients with CVD were subjected to vitamin D deficiency (serum 25[OH]D <50 nmol/L). Among patients with existing CVD, increasing levels in serum 25(OH)D were independently associated with a decreased risk of all-cause and cause-specific mortality. Such associations presented non-linear trends for all-cause, cancer, respiratory disease, and other-cause mortality, and a linear trend for cardiovascular mortality. Our findings underscore the importance of optimizing vitamin D status for patients with existing CVD, especially for those subjected to vitamin D deficiency.

Although numerous observational studies have examined the association of vitamin D with all-cause and cause-specific mortality, most studies focused on the general population and deliberately excluded patients with known CVD (37). Existing evidence for the long-term association between vitamin D status and adverse outcomes in CVD patients is inconsistent and insufficient (20–27). One cohort study including 1,125 German patients with stable CHD found no significant association of serum 25 (OH)D levels with secondary cardiovascular event incidence and all-cause mortality (27). In another longitudinal study with 946 stable CHD patients in the San Francisco Bay Area, Welles et al. found that 25(OH)D levels under 50 nmol/L remain independently associated with cardiovascular events, but the independent association is no longer present (adjusted HR, 1.11 [95% CI: 0.85, 1.44]) after further adjustment for potential biological mediators [i.e., blood pressure, lipids, HbA1c, CRP, and PTH; (26)]. Three small-sample studies found that vitamin D deficiency is associated with an increased risk of ischemic heart disease events among 244 patients with prior CVD (including ischemic heart disease, peripheral vascular disease, and stroke) (20). In addition, a low 25(OH)D concentration is associated with a poor prognosis in patients with HF (*n* = 148 and *n* = 548) (22, 23). A clinic-based study in Israel found that vitamin D deficiency is more prevalent in HF patients (*n* = 3,009) than in the control group (*n* = 46,825) (21). HF patients with severe deficiency of vitamin D (25[OH]D <25.0 nmol/L) have higher risk of mortality than those with serum 25(OH)D ≥75.0 nmol/L (adjusted HR, 1.61 [95% CI: 1.08, 2.41]). The authors observed that vitamin D supplementation is associated with reduced mortality in HF patients with an (adjusted HR, 0.68 [95% CI: 0.54–0.85]). However, the clinic-based design and relatively short follow-up duration (median 518 days) might limit the generalizability of these findings to the broader population of CVD patients and long-term outcomes. Similar limitations also remained in the hospital-based single-center study comprising 3,316 patients of White ethnicity referred for coronary angiography (24). They also had no clear diagnoses of CVD and subtypes. In another study among 4,114 white patients suspected of having stable angina pectoris, Degerud et al. found that plasma 25(OH)D concentrations are non-linearly (U-shaped) associated with all-cause mortality; near 70 nmol/L 25(OH)D corresponds to the lowest mortality risk, and their analysis suggested increased all-cause mortality at concentrations >100 nmol/L (25). However, our findings were not consistent with this trend. All-cause mortality decreased with increasing serum 25(OH)D concentration and then reached a plateau at around 50 nmol/L 25(OH)D. A small amount could potentially decrease the risk of cardiovascular mortality with higher 25(OH)D concentrations (>100 nmol/L) in our analyses. This continuing downward trend of all-cause and cardiovascular mortality was more pronounced among patients with stroke. Further clinical trials are warranted to determine the benefits of vitamin D supplementation among patients with existing CVD and subtypes, and the threshold range of 25(OH)D is applicable to vitamin D supplementation.

To the best of our knowledge, this dataset is the largest to examine the associations of serum 25(OH)D concentrations with all-cause and cause-specific mortality among adult patients with existing CVD. Compared with previous studies, the present study considered much more comprehensive confounders, conducted numerous sensitivity analyses, and included various CVD subtypes. Higher serum 25(OH)D levels were significantly associated with lower all-cause and cause-specific mortality among patients with CVD. Such associations presented non-linear trends for all-cause, cancer, respiratory disease, and other-cause mortality and an approximately linear trend for cardiovascular mortality. These results suggest that CVD patients with vitamin D deficiency (25[OH]D <50 nmol/L) are more likely to benefit from optimizing vitamin D status than those without vitamin D deficiency. An interesting finding was that the analysis of patients with stroke showed impressive reductions in CVD mortality for the group with sufficient vitamin D (25[OH]D ≥75.0 nmol/L), with an adjusted HR of 0.45 (95% CI: 0.29, 0.70). This finding implied that stroke patients might derive additional cardiovascular benefits from maintaining more adequate vitamin D status. Although insufficient evidence has been proposed in this regard, our findings provided a novel perspective of vitamin D status and cardiovascular morbidity and mortality in patients with stroke. Nowadays, several RCTs are underway to assess the effect of vitamin D supplementation on patients with heart failure (i.e., NCT03416361, NCT03289637), idiopathic cardiomyopathies (i.e., NCT02517814), and coronary artery disease (i.e., NCT01570309, NCT02996721). In the future, these clinical trials may further demonstrate our findings.

Several possible mechanisms could be involved in explaining the effects of vitamin D in CVD. Vitamin D receptors (VDRs) are widely expressed in the cardiovascular system (cardiomyocytes, vascular endothelial cells, vascular smooth muscle cells, fibroblasts, pericytes, platelets, macrophages, and other immune cells), and activated vitamin D binding to VDRs regulates multiple cardiovascular mechanisms (2). In the blood vessels, vitamin D may modulate vascular tone via regulation of calcium influx and stimulation of nitric oxide production in smooth muscle cells (38, 39). In addition, it also exhibits anti-inflammatory actions, inhibition of foam cell formation, and reduction of thrombogenicity and vascular calcifications (39, 40). Vitamin D deficiency accelerates CAD progression by increasing karyopherin α4 and nuclear factor kappa beta levels (41). In the heart, vitamin D treatment may inhibit cardiac hypertrophy, modulate cardiac contractility, regulate extracellular matrix turnover, and attenuate left ventricular abnormalities (42, 43). The cardiovascular system appears to be highly sensitive to vitamin D deficiency (38). In humans, vitamin D deficiency could be linked to vascular dysfunction, arterial stiffness, reduced coronary flow, subclinical atherosclerosis, and left ventricular hypertrophy (44, 45). All the mentioned studies demonstrate that vitamin D treatment could have salutary effects in the development of CVD. Given that the mechanism of whether and how vitamin D deficiency exacerbates the progression of CVD is limited, additional studies are warranted to clarify the potential mechanisms.

The strengths of our study include the large number of participants and the wide range of serum 25(OH)D levels. The high-quality data from UK Biobank favor the definition and classification of various CVD subtypes and allow us to examine the long-term association of vitamin D status with all-cause and cause-specific mortality. Associations were robust after adjustment for a wide range of potential confounding factors, including socioeconomic status, dietary and lifestyle factors, comorbidities, and CVD duration. In addition, the robustness of these findings was confirmed by comprehensive sensitivity analyses. Several potential limitations need to be considered. First, while our research cannot make a causal inference because of the observational nature, it provides evidence to support the importance of conducting future studies using a more rigorous study design, including RCTs. Second, although we considered a wide range of covariates and performed adequate sensitivity analyses, residual confounders and potential bias may still be present. Third, since the UK Biobank cohort included the largely white population, the results from this study may not be directly generalizable to other populations. Fourth, we were unable to access the relationship between dynamic changes in 25(OH)D concentrations and mortality because the 25(OH)D measurements were not repeated. Additionally, a single measurement of serum 25(OH)D might not represent long-term vitamin D status, but 25(OH)D values do appear to remain relatively stable over time (46). Fifth, although the competing risk of cancer death existed in CVD patients, it appeared to be a minor effect, and the cause-specific hazard function model was used in our cox regression analyses.

## Conclusion

In the UK Biobank, nearly 60% of CVD patients were subjected to vitamin D deficiency. Among patients with existing CVD, increasing levels in serum 25(OH)D were independently associated with a decreased risk of all-cause and cause-specific mortality in a non-linear and dose-response manner. Compared with CVD patients with serum 25(OH)D ≥50 nmol/L, those subjected to vitamin D deficiency benefited more from an increment in serum 25(OH)D. Our findings provided novel clues awaiting further validation in clinical trials.

## Data Availability Statement

The original contributions presented in the study are included in the article/Supplementary Material, further inquiries can be directed to the corresponding author/s.

## Ethics Statement

The studies involving human participants were reviewed and approved by ethical approval was obtained by the National Health Service National Research Ethics Service (11/NW/0382) and renewed by the North West–Haydock Research Ethics Committee (16/NW/0274). All participants provided informed written consent. The background information about UK Biobank and details is available on the website http://www.ukbiobank.ac.uk. The patients/participants provided their written informed consent to participate in this study.

## Author Contributions

LC conceived and designed the study, revised it critically for important intellectual content, and attests that all listed authors meet authorship criteria and that no others meeting the criteria have been omitted. LD and ML analyzed and interpreted data. LD drafted the manuscript. All authors provided final approval of the version to be published.

## Funding

LC was supported by the National Key Research and Development Program of China (2020YFC2006300) and the National Natural Science Foundation of China (82003461). The funders had no role in study design, data collection and analysis, decision to publish, or preparation of the manuscript.

## Conflict of Interest

The authors declare that the research was conducted in the absence of any commercial or financial relationships that could be construed as a potential conflict of interest.

## Publisher's Note

All claims expressed in this article are solely those of the authors and do not necessarily represent those of their affiliated organizations, or those of the publisher, the editors and the reviewers. Any product that may be evaluated in this article, or claim that may be made by its manufacturer, is not guaranteed or endorsed by the publisher.
