# Supplementary material for: Association of Serum 25-Hydroxyvitamin D Concentrations With All-Cause and Cause-Specific Mortality Among Adult Patients With Existing Cardiovascular Disease
Source: Front Nutr. 2021 Sep 23;8:740855. doi: 10.3389/fnut.2021.740855 (PMC8496747; doi:10.3389/fnut.2021.740855)
Supplement: Supplementary file 1 [file Data_Sheet_1.docx]

**Supplementary Online Content**

Supplement Table 1. Cardiovascular disease definitions in UK Biobank study

Supplement Table 2. Definition of each component of a healthy diet score

Supplement Table 3. Stratified analyses of the associations of serum 25(OH)D concentrations with all-cause and cardiovascular mortality

Supplement Table 4. Sensitivity analyses of the associations of serum 25(OH)D concentrations with all-cause and cause-specific mortality among 37,079 CVD patients

Supplement Table 5. Hazards of all-cause and cause-specific mortality with further adjustment of serum calcium and phosphate after excluding participants with history of thyroid or parathyroid diseases (n=33,973)

Supplement Table 6. Hazards of all-cause and cause-specific mortality after excluding participants who died within four years of follow-up (n=35,721)

Supplement Table 7. Hazards of all-cause and cause-specific mortality with further adjustment of energy intake and dietary vitamin D intake (n=12,505)

Supplement Figure 1. Flowchart of eligible population

Supplement Figure 2. Associations of serum 25(OH)D concentrations with cancer, respiratory disease, and other-cause mortality

Supplementary Figure 3. Associations between serum 25(OH)D concentrations with time spend outdoors and month of blood collection

**Supplement Table 1. Cardiovascular disease definitions in UK Biobank study**

|  | **ICD-1** | **ICD-10** | **OPCS-4** | **Self-reported field IDs** |
| --- | --- | --- | --- | --- |
| **Coronary Heart Disease** | 410, 411, 412, 413, 414 | I20-25 | K40-K46, K49, K50, K75 | 6150, 3894, 3637, 2002, 20004 |
| **Heart Failure** | 428, 4280, 4281,4289 | I50, I500, I501, I509 |  | 20002 |
| **Atrial Fibrillation** | 4273 | I48 | K621, K622, K623 | 20002 |
| **Stroke** | 430, 431, 434, 4340, 4341, 4349, 436 | I60, I61, I63, I64 | A052-A054, L351, L353, L343 | 6150, 4056, 20002 |

Variable definitions constructed using ICD-9, ICD-10 and OPCS-4 codes as well as self-reported data fields with choice-, disease- or procedure-specific codes between brackets are shown.

Abbreviations: ICD, International Classification of Diseases; OPCS, Office of Population, Censuses and Surveys: Classification of interventions and Procedures.

**Supplement Table 2. Definition of each component of a healthy diet score**

|  | **Goal (1 point)** | **One serving equals to** | **Field IDs** |
| --- | --- | --- | --- |
| **Fruits** | ≥ 3 servings/day | 1 piece of fresh fruit  5 pieces of dried fruit | 1309, 1319 |
| **Vegetables (excluding potatoes)** | ≥ 3 servings/day | 3 heaped tablespoons | 1289, 1299 |
| **Whole grains** | ≥ 3 servings/day | 1 slice of whole-grain bread  1 cup of whole-grain cereal | 1438, 1448, 1458, 1468 |
| **Vegetable oil** | ≥ 2 servings/day | Vegetable oil based spread (Flora Pro-Active/Benecol, Soft (tub) margarine, Olive oil based spread, or Polyunsaturated/sunflower oil based spread) in combination with eating at least 2 slices of bread | 1428, 2654, 1438 |
| **Fish** | ≥ 2 servings/week | Once/week | 1329, 1339 |
| **Dairy** | ≥ 2 servings/day | 1 cup/day if consumption any type of milk  1 piece of cheese | 1408, 1418 |
| **Refined grains** | ≤ 2 servings/day | 1 slice of bread or 1 bowl of cereal | 1438, 1448, 1458, 1468 |
| **Unprocessed meats** | ≤ 2 servings/week | Once/week (including poultry, beef, lamb, and pork) | 1359, 1369, 1379, 1389 |
| **Processed meats** | ≤ 1 servings/week | Once/week | 1349 |
| **Sugar-sweetened beverages** | Don’t drink | Only 0 serving was possible here | 6144 |

Field IDs and serving sizes used per diet component in UK Biobank with available data from the general baseline questionnaire. If participants achieved the intake goal they were considered to have an adequate intake of the diet component. Adequate intake of at least half of all diet components was considered as an ideal diet, less than half was considered a poor diet.

**Supplement Table 3. Stratified analyses of the associations of serum 25(OH)D concentrations with all-cause and cardiovascular mortality**

|  | **Serum 25(OH)D concentrations** | | | | |
| --- | --- | --- | --- | --- | --- |
|  | **<25.0 nmol/L** | **25.0–49.9 nmol/L** | **50.0–74.9 nmol/L** | **≥75.0 nmol/L** | ***P* value for interaction** |
| **All-cause mortality** |  |  |  |  |  |
| **Age, years** |  |  |  |  |  |
| <60 (n=11,082) | 1 (ref.) | 0.81 (0.70, 0.93) | 0.74 (0.62, 0.89) | 0.53 (0.39, 0.71) | 0.27 |
| ≥60 (n=25,998) | 1 (ref.) | 0.81 (0.75, 0.88) | 0.74 (0.68, 0.81) | 0.71 (0.64, 0.79) |  |
| **Sex** |  |  |  |  |  |
| Male (n=24,412) | 1 (ref.) | 0.78 (0.72, 0.85) | 0.71 (0.65, 0.78) | 0.64 (0.57, 0.72) | 0.79 |
| Female (n=12,668) | 1 (ref.) | 0.78 (0.69, 0.89) | 0.67 (0.58, 0.78) | 0.72 (0.59, 0.88) |  |
| **BMI, kg/m^2^** |  |  |  |  |  |
| <30.0 (n=23,201) | 1 (ref.) | 0.73 (0.67, 0.80) | 0.67 (0.61, 0.74) | 0.64 (0.56, 0.72) | 0.33 |
| ≥30.0 (n=13,579) | 1 (ref.) | 0.81 (0.73, 0.89) | 0.71 (0.63, 0.80) | 0.67 (0.55, 0.81) |  |
| **Smoking status, n (%)** |  |  |  |  |  |
| Never smoker (n=15,100) | 1 (ref.) | 0.83 (0.72, 0.95) | 0.71 (0.61, 0.83) | 0.75 (0.62, 0.91) | 0.88 |
| Former smoker (n=17,242) | 1 (ref.) | 0.75 (0.68, 0.83) | 0.66 (0.60, 0.74) | 0.60 (0.52, 0.69) |  |
| Current smoker (n=4,433) | 1 (ref.) | 0.82 (0.72, 0.93) | 0.81 (0.69, 0.96) | 0.69 (0.54, 0.90) |  |
| **Physical activity, n (%)** |  |  |  |  |  |
| Inactive group (n=7,469) | 1 (ref.) | 0.85 (0.75, 0.96) | 0.73 (0.63, 0.84) | 0.83 (0.68, 1.02) | 0.076 |
| Insufficient group (n=9,589) | 1 (ref.) | 0.78 (0.68, 0.89) | 0.66 (0.57, 0.77) | 0.60 (0.48, 0.75) |  |
| Active group (n=17,033） | 1 (ref.) | 0.73 (0.65, 0.83) | 0.70 (0.62, 0.80) | 0.63 (0.53, 0.73) |  |
| **Dietary supplement use** |  |  |  |  |  |
| Yes (n=18,198) | 1 (ref.) | 0.76 (0.68, 0.85) | 0.67 (0.60, 0.76) | 0.66 (0.57, 0.76) | 0.74 |
| No (n= 18,882) | 1 (ref.) | 0.79 (0.72, 0.86) | 0.71 (0.64, 0.79) | 0.61 (0.52, 0.72) |  |
| **Antihypertensive treatment** |  |  |  |  |  |
| Yes (n=22,444) | 1 (ref.) | 0.81 (0.75, 0.88) | 0.73 (0.66, 0.80) | 0.66 (0.59, 0.75) | 0.23 |
| No (n= 14,636) | 1 (ref.) | 0.72 (0.64, 0.81) | 0.64 (0.56, 0.74) | 0.62 (0.52, 0.74) |  |
| **Cholesterol lowering medication** |  |  |  |  |  |
| Yes (n=26,636) | 1 (ref.) | 0.79 (0.73, 0.85) | 0.70 (0.64, 0.77) | 0.65 (0.58, 0.73) | 0.85 |
| No (n= 10,444) | 1 (ref.) | 0.77 (0.67, 0.89) | 0.68 (0.58, 0.80) | 0.67 (0.53, 0.83) |  |
| **Duration of CVD, years** |  |  |  |  |  |
| <5.0 (n=15,883) | 1 (ref.) | 0.77 (0.69, 0.87) | 0.70 (0.62, 0.80) | 0.65 (0.55, 0.76) | 0.56 |
| 5.0-9.9 (n=10,059) | 1 (ref.) | 0.79 (0.69, 0.90) | 0.72 (0.62, 0.83) | 0.63 (0.51, 0.77) |  |
| ≥10.0 (n=11,138) | 1 (ref.) | 0.78 (0.70, 0.87) | 0.68 (0.60, 0.77) | 0.67 (0.57, 0.79) |  |
|  |  |  |  |  |  |
| **Cardiovascular mortality** |  |  |  |  |  |
| **Age, years** |  |  |  |  |  |
| <60 (n=11,082) | 1 (ref.) | 0.75 (0.60, 0.95) | 0.71 (0.53, 0.96) | 0.38 (0.22, 0.67) | 0.19 |
| ≥60 (n=25,998) | 1 (ref.) | 0.82 (0.72, 0.93) | 0.74 (0.64, 0.86) | 0.63 (0.52, 0.77) |  |
| **Sex** |  |  |  |  |  |
| Male (n=24,412) | 1 (ref.) | 0.79 (0.69, 0.90) | 0.74 (0.64, 0.86) | 0.61 (0.50, 0.75) | 0.26 |
| Female (n=12,668) | 1 (ref.) | 0.83 (0.65, 1.04) | 0.61 (0.46, 0.81) | 0.49 (0.32, 0.74) |  |
| **BMI, kg/m^2^** |  |  |  |  |  |
| <30.0 (n=23,201) | 1 (ref.) | 0.74 (0.63, 0.87) | 0.67 (0.56, 0.80) | 0.53 (0.42, 0.67) | 0.14 |
| ≥30.0 (n=13,579) | 1 (ref.) | 0.80 (0.68, 0.94) | 0.72 (0.60, 0.88) | 0.69 (0.51, 0.93) |  |
| **Smoking status, n (%)** |  |  |  |  |  |
| Never smoker (n=15,100) | 1 (ref.) | 0.70 (0.55, 0.87) | 0.61 (0.48, 0.79) | 0.62 (0.44, 0.87) | 0.35 |
| Former smoker (n=17,242) | 1 (ref.) | 0.77 (0.65, 0.90) | 0.67 (0.56, 0.80) | 0.53 (0.41, 0.68) |  |
| Current smoker (n=4,433) | 1 (ref.) | 0.96 (0.77, 1.21) | 0.98 (0.73, 1.31) | 0.62 (0.37, 1.02) |  |
| **Physical activity, n (%)** |  |  |  |  |  |
| Inactive group (n=7,469) | 1 (ref.) | 0.83 (0.67, 1.01) | 0.69 (0.54, 0.89) | 0.60 (0.41, 0.89) | 0.65 |
| Insufficient group (n=9,589) | 1 (ref.) | 0.88 (0.70, 1.10) | 0.73 (0.56, 0.95) | 0.59 (0.40, 0.86) |  |
| Active group (n=17,033） | 1 (ref.) | 0.71 (0.58, 0.87) | 0.68 (0.54, 0.84) | 0.57 (0.43, 0.75) |  |
| **Dietary supplement use** |  |  |  |  |  |
| Yes (n=18,198) | 1 (ref.) | 0.78 (0.65, 0.94) | 0.67 (0.54, 0.82) | 0.59 (0.46, 0.76) | 0.92 |
| No (n= 18,882) | 1 (ref.) | 0.80 (0.69, 0.92) | 0.77 (0.64, 0.92) | 0.54 (0.41, 0.72) |  |
| **Antihypertensive treatment** |  |  |  |  |  |
| Yes (n=22,444) | 1 (ref.) | 0.83 (0.73, 0.95) | 0.76 (0.65, 0.88) | 0.59 (0.48, 0.74) | 0.26 |
| No (n= 14,636) | 1 (ref.) | 0.69 (0.55, 0.86) | 0.62 (0.48, 0.79) | 0.54 (0.39, 0.76) |  |
| **Cholesterol lowering medication** | |  |  |  |  |
| Yes (n=26,636) | 1 (ref.) | 0.80 (0.71, 0.91) | 0.70 (0.61, 0.81) | 0.59 (0.48, 0.72) | 0.62 |
| No (n= 10,444) | 1 (ref.) | 0.77 (0.58, 1.03) | 0.75 (0.55, 1.02) | 0.59 (0.37, 0.93) |  |
| **Duration of CVD, years** |  |  |  |  |  |
| <5.0 (n=15,883) | 1 (ref.) | 0.81 (0.65, 0.99) | 0.67 (0.53, 0.85) | 0.60 (0.43, 0.83) | 0.34 |
| 5.0-9.9 (n=10,059) | 1 (ref.) | 0.77 (0.61, 0.97) | 0.82 (0.63, 1.05) | 0.52 (0.35, 0.75) |  |
| ≥10.0 (n=11,138) | 1 (ref.) | 0.81 (0.68, 0.96) | 0.69 (0.56, 0.84) | 0.61 (0.46, 0.80) |  |

Data were presented as hazard ratios (95% CIs) with adjustment of age, sex, and ethnicity, education, Townsend deprivation index, household income, smoking status, alcohol consumption, physical activity, healthy diet score, BMI, eGFRcr-cys, C-reactive protein, antihypertensive medication use, cholesterol lowering medication use, diabetes medication use, history of cancer, diabetes, hypertension, and duration of CVD.

Abbreviations: BMI, body mass index; CVD, cardiovascular disease; eGFRcr-cys, estimated glomerular filtration rate (creatinine–cystatin C equation).

**Supplement Table 4. Sensitivity analyses of the associations of serum 25(OH)D concentrations with all-cause and cause-specific mortality among 37,079 CVD patients**

|  | **Serum 25(OH)D concentrations, nmol/L** | | | |
| --- | --- | --- | --- | --- |
|  | **<25.0 nmol/L (n=5,773)** | **25.0–49.9 nmol/L (n=15,557)** | **50.0–74.9 nmol/L (n=11,451)** | **≥75.0 nmol/L (n=4,298)** |
| **All-cause mortality** |  |  |  |  |
| Model 1 | 1 (ref.) | 0.76 (0.71, 0.81) | 0.66 (0.61, 0.72) | 0.61 (0.55, 0.68) |
| Model 2 | 1 (ref.) | 0.76 (0.71, 0.81) | 0.66 (0.61, 0.71) | 0.60 (0.54, 0.67) |
| Model 3 | 1 (ref.) | 0.75 (0.70, 0.80) | 0.64 (0.59, 0.70) | 0.57 (0.51, 0.64) |
| **Cardiovascular disease mortality** |  |  |  |  |
| Model 1 | 1 (ref.) | 0.76 (0.68, 0.85) | 0.66 (0.57, 0.75) | 0.53 (0.44, 0.64) |
| Model 2 | 1 (ref.) | 0.76 (0.68, 0.85) | 0.65 (0.57, 0.75) | 0.52 (0.43, 0.63) |
| Model 3 | 1 (ref.) | 0.74 (0.66, 0.83) | 0.63 (0.55, 0.73) | 0.48 (0.40, 0.59) |
| **Cancer mortality** |  |  |  |  |
| Model 1 | 1 (ref.) | 0.88 (0.78, 0.997) | 0.78 (0.68, 0.89) | 0.76 (0.64, 0.91) |
| Model 2 | 1 (ref.) | 0.89 (0.79, 1.004) | 0.79 (0.69, 0.91) | 0.78 (0.65, 0.93) |
| Model 3 | 1 (ref.) | 0.89 (0.79, 1.01) | 0.79 (0.69, 0.92) | 0.78 (0.65, 0.94) |
| **Respiratory disease mortality** |  |  |  |  |
| Model 1 | 1 (ref.) | 0.70 (0.57, 0.87) | 0.54 (0.42, 0.70) | 0.59 (0.42, 0.82) |
| Model 2 | 1 (ref.) | 0.71 (0.58, 0.88) | 0.55 (0.42, 0.71) | 0.59 (0.42, 0.82) |
| Model 3 | 1 (ref.) | 0.70 (0.57, 0.87) | 0.54 (0.41, 0.70) | 0.55 (0.39, 0.77) |
| **Other mortality** |  |  |  |  |
| Model 1 | 1 (ref.) | 0.63 (0.54, 0.73) | 0.57 (0.48, 0.68) | 0.55 (0.43, 0.69) |
| Model 2 | 1 (ref.) | 0.62 (0.53, 0.72) | 0.55 (0.46, 0.65) | 0.51 (0.41, 0.65) |
| Model 3 | 1 (ref.) | 0.61 (0.52, 0.70) | 0.53 (0.44, 0.63) | 0.47 (0.37, 0.60) |

Model 1: adjusted for the main model (age, sex, ethnicity, education, Townsend deprivation index, household income, smoking status, alcohol consumption, physical activity, healthy diet score, BMI, eGFRcr-cys, C-reactive protein, antihypertensive medication use, cholesterol lowering medication use, diabetes medication use, history of cancer, diabetes, hypertension, and duration of CVD) plus month of blood collection (January through December, categorical), time spend outdoors in summer (continuous), and time spend outdoors in winter (continuous).

Model 2: adjusted for model 1 plus dietary supplement use, including vitamin supplements (yes/no), including vitamin D supplements (yes/no), multivitamin supplements (yes/no), mineral supplements (yes/no), fish oil (yes/no), and glucosamine (yes/no).

Model 3: adjusted for model 2 plus mean arterial pressure (in quintiles), hemoglobin A1c (in quintiles), triglycerides (in quintiles), high density lipoprotein cholesterol (in quintiles)

Abbreviations: BMI, body mass index; CVD, cardiovascular disease; eGFRcr-cys, estimated glomerular filtration rate (creatinine–cystatin C equation).

**Supplement Table 5. Hazards of all-cause and cause-specific mortality with further adjustment of serum calcium and phosphate after excluding participants with history of thyroid or parathyroid diseases (n=33,973)**

|  | **Serum 25(OH)D concentrations, nmol/L** | | | |
| --- | --- | --- | --- | --- |
|  | **<25.0 nmol/L  (n=5,205)** | **25.0-49.9 nmol/L  (n=14,234)** | **50.0-74.9 nmol/L  (n=10,563)** | **≥75.0 nmol/L  (n=3,971)** |
| **All-cause mortality** | 1 (ref.) | 0.78 (0.73, 0.84) | 0.70 (0.65, 0.76) | 0.66 (0.60, 0.74) |
| **Cardiovascular disease mortality** | 1 (ref.) | 0.80 (0.71, 0.90) | 0.73 (0.64, 0.84) | 0.59 (0.49, 0.72) |
| **Cancer mortality** | 1 (ref.) | 0.87 (0.77, 0.99) | 0.78 (0.68, 0.89) | 0.77 (0.65, 0.92) |
| **Respiratory disease mortality** | 1 (ref.) | 0.70 (0.56, 0.87) | 0.56 (0.44, 0.73) | 0.61 (0.44, 0.86) |
| **Other mortality** | 1 (ref.) | 0.68 (0.59, 0.80) | 0.64 (0.53, 0.76) | 0.64 (0.51, 0.81) |

Data were presented as hazard ratios (95% CIs) with adjustment of the main model (age, sex, ethnicity, education, Townsend deprivation index, household income, smoking status, alcohol consumption, physical activity, healthy diet score, BMI, eGFRcr-cys, C-reactive protein, antihypertensive medication use, cholesterol lowering medication use, diabetes medication use, history of cancer, diabetes, hypertension, and duration of CVD) plus serum calcium concentrations (in quintiles) and serum phosphate concentrations (in quintiles).

Abbreviations: BMI, body mass index; CVD, cardiovascular disease; eGFRcr-cys, estimated glomerular filtration rate (creatinine–cystatin C equation).

**Supplement Table 6. Hazards of all-cause and cause-specific mortality after excluding participants who died within four years of follow-up (n=35,721)**

|  | **Serum 25(OH)D concentrations, nmol/L** | | | |
| --- | --- | --- | --- | --- |
|  | **<25.0 nmol/L  (n=5,456)** | **25.0-49.9 nmol/L  (n=14,966)** | **50.0-74.9 nmol/L  (n=11,123)** | **≥75.0 nmol/L  (n=4,176)** |
| **All-cause mortality** | 1 (ref.) | 0.78 (0.72, 0.84) | 0.71 (0.65, 0.77) | 0.66 (0.59, 0.74) |
| **Cardiovascular disease mortality** | 1 (ref.) | 0.81 (0.71, 0.92) | 0.74 (0.64, 0.86) | 0.57 (0.46, 0.71) |
| **Cancer mortality** | 1 (ref.) | 0.86 (0.75, 0.99) | 0.80 (0.69, 0.93) | 0.76 (0.62, 0.92) |
| **Respiratory disease mortality** | 1 (ref.) | 0.70 (0.56, 0.87) | 0.56 (0.43, 0.73) | 0.63 (0.45, 0.88) |
| **Other mortality** | 1 (ref.) | 0.68 (0.58, 0.80) | 0.64 (0.54, 0.77) | 0.67 (0.53, 0.84) |

Data were presented as hazard ratios (95% CIs) with adjustment of the main model (age, sex, ethnicity, education, Townsend deprivation index, household income, smoking status, alcohol consumption, physical activity, healthy diet score, BMI, eGFRcr-cys, C-reactive protein, antihypertensive medication use, cholesterol lowering medication use, diabetes medication use, history of cancer, diabetes, hypertension, and duration of CVD)

Abbreviations: BMI, body mass index; CVD, cardiovascular disease; eGFRcr-cys, estimated glomerular filtration rate (creatinine–cystatin C equation).

**Supplement Table 7. Hazards of all-cause and cause-specific mortality with** **further adjustment of energy intake and dietary vitamin D intake (n=12,505*)**

|  | **Serum 25(OH)D concentrations, nmol/L** | | | |
| --- | --- | --- | --- | --- |
|  | **<25.0 nmol/L  (n=1,448)** | **25.0-49.9 nmol/L  (n=5,129)** | **50.0-74.9 nmol/L  (n=4,251)** | **≥75.0 nmol/L  (n=1,677)** |
| **All-cause mortality** | 1 (ref.) | 0.79 (0.68, 0.92) | 0.70 (0.59, 0.82) | 0.69 (0.56, 0.85) |
| **Cardiovascular disease mortality** | 1 (ref.) | 0.74 (0.58, 0.96) | 0.58 (0.44, 0.77) | 0.57 (0.39, 0.81) |
| **Cancer mortality** | 1 (ref.) | 1.002 (0.76, 1.31) | 0.84 (0.63, 1.13) | 0.93 (0.66, 1.31) |
| **Respiratory disease mortality** | 1 (ref.) | 1.03 (0.60, 1.79) | 1.001 (0.56, 1.79) | 0.75 (0.36, 1.56) |
| **Other mortality** | 1 (ref.) | 0.53 (0.38, 0.74) | 0.59 (0.41, 0.84) | 0.55 (0.35, 0.87) |

*Dietary information was collected through a web based 24 hour recall questionnaire ([www.ceu.ox.ac.uk/research/oxford-webq](http://www.ceu.ox.ac.uk/research/oxford-webq)). UK Biobank participants (n=211,014) were invited to complete one to five times dietary recalls between April 2009 and June 2012. Nutrient intake was estimated using *McCance and Widdowson’s Composition of Foods* (the fifth edition) and we calculated mean values from the available data (see reference: BMJ. 2020;368:m688). Among 37080 patients with CVD, only 12505 of them completed the dietary recalls.

Data were presented as hazard ratios (95% CIs) with adjustment of the main model (age, sex, ethnicity, education, Townsend deprivation index, household income, smoking status, alcohol consumption, physical activity, healthy diet score, BMI, eGFRcr-cys, C-reactive protein, antihypertensive medication use, cholesterol lowering medication use, diabetes medication use, history of cancer, diabetes, hypertension, and duration of CVD) plus total energy intake (continuous) and dietary vitamin D intake (continuous).

Abbreviations: BMI, body mass index; CVD, cardiovascular disease; eGFRcr-cys, estimated glomerular filtration rate (creatinine–cystatin C equation).


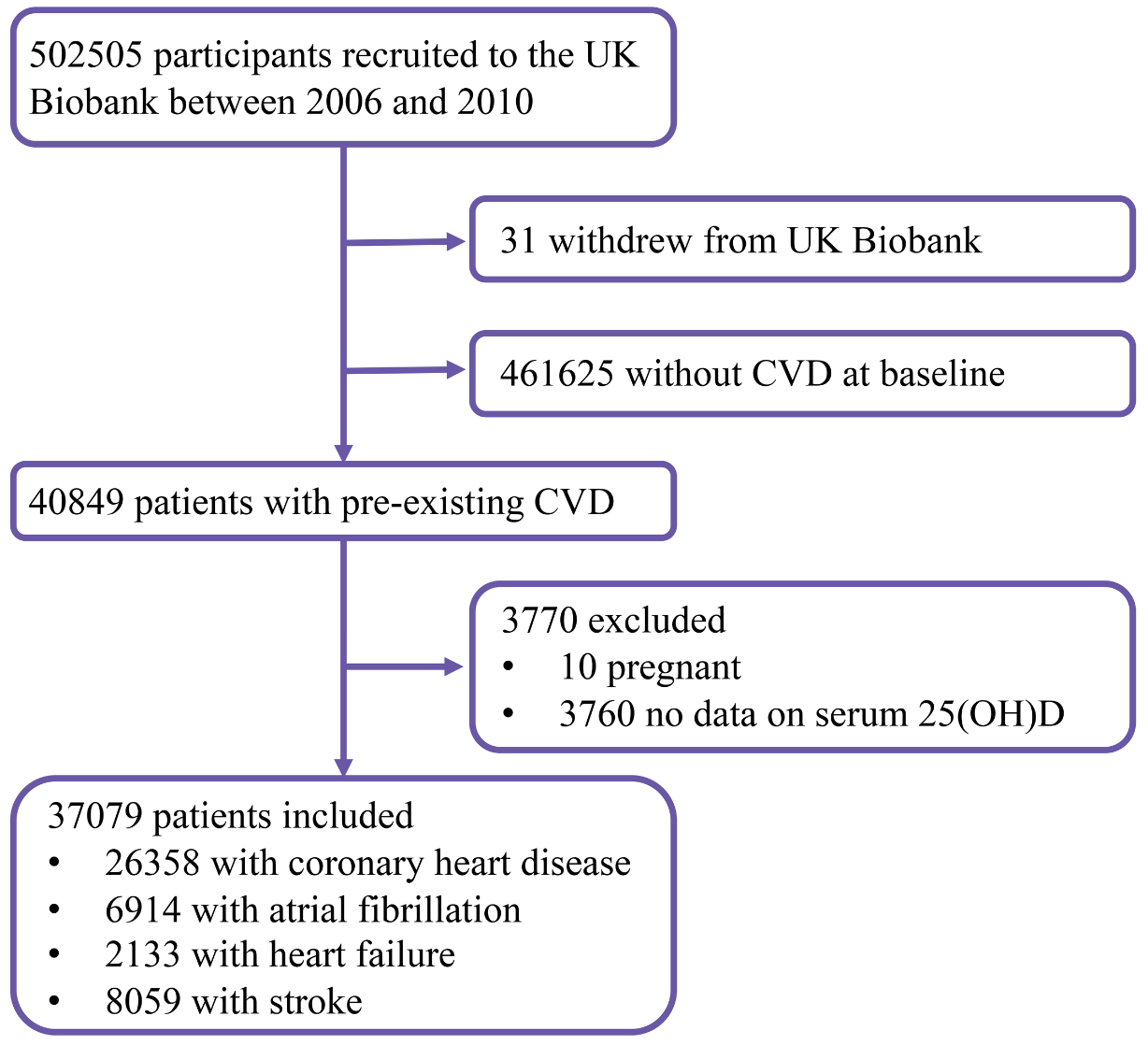


**Supplement Figure 1. Flowchart of eligible population.**


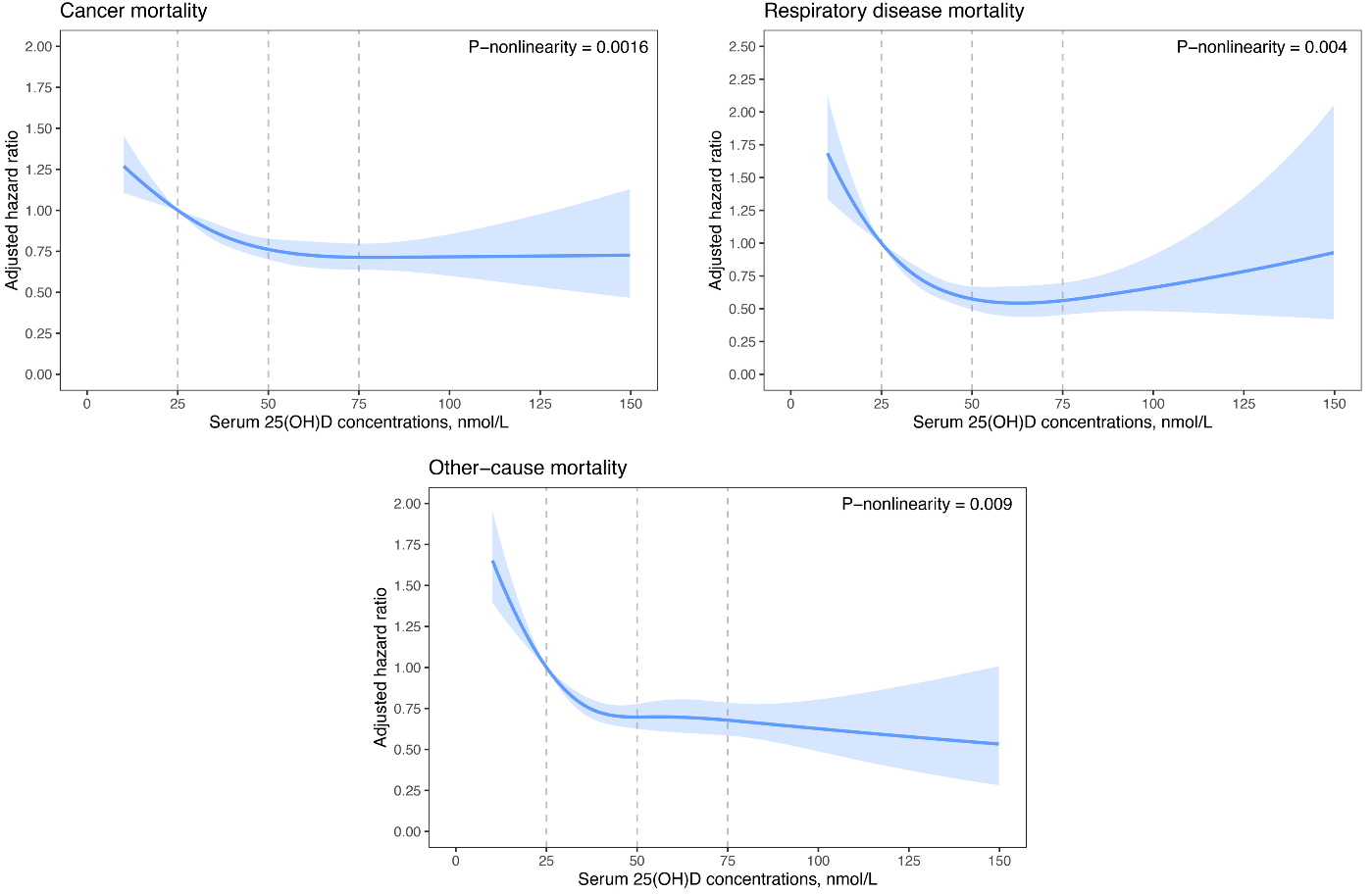


**Supplement Figure 2. Associations of serum 25(OH)D concentrations with cancer, respiratory disease, and other-cause mortality**

Hazard ratios (blue lines) and 95% confidence intervals (light blue shade) were adjusted for age, sex, and ethnicity, education, Townsend deprivation index, household income, smoking status, alcohol consumption, physical activity, healthy diet score, BMI, eGFRcr-cys, C-reactive protein, antihypertensive medication use, cholesterol lowering medication use, diabetes medication use, history of cancer, diabetes, hypertension, and duration of CVD.

Abbreviations: BMI, body mass index; CVD, cardiovascular disease; eGFRcr-cys, estimated glomerular filtration rate (creatinine–cystatin C equation).

**
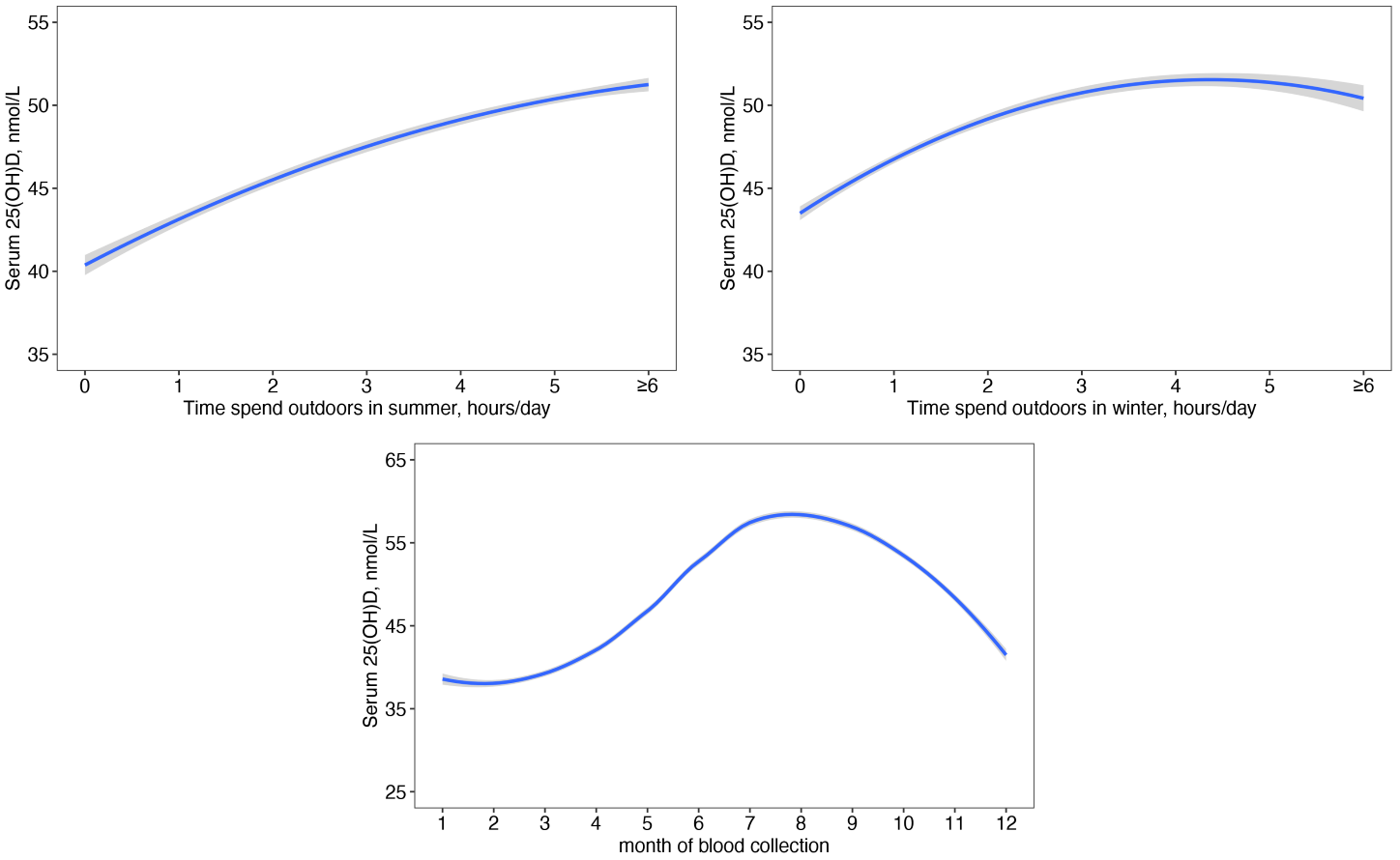
Supplementary Figure 3. Associations between serum 25(OH)D concentrations with time spend outdoors and month of blood collection**

The associations of time spend outdoors in summer and time spend outdoors in winter with serum 25(OH)D concentrations were presented by using generalized additive models. The seasonal changes in serum 25(OH)D were presented using locally weighted scatterplot smoothing models.
